# How to Define and Manage Low-Risk Drug Allergy Labels

**DOI:** 10.1016/j.jaip.2024.03.021

**Published:** 2024-05

**Authors:** Ana Maria Copaescu, Lily Li, Kimberly G. Blumenthal, Jason A. Trubiano

**Affiliations:** a Centre for Antibiotic Allergy and Research, Department of Infectious Diseases, Austin Health, Heidelberg, VIC, Australia; b Department of Medicine, Austin Health, the University of Melbourne, Heidelberg, VIC, Australia; c Division of Allergy and Clinical Immunology, Department of Medicine, McGill University Health Centre (MUHC), McGill University, Montreal, QC, Canada; d The Research Institute of the McGill University Health Centre, McGill University Health Centre (MUHC), McGill University, Montreal, QC, Canada; e Division of Allergy and Infectious Diseases, Department of Medicine, University of Washington, Seattle, Wash; f Division of Rheumatology, Allergy, and Immunology, Department of Medicine, Massachusetts General Hospital, Boston, Mass; g Harvard Medical School, Boston, Mass; h Department of Infectious Diseases, University of Melbourne at the Peter Doherty Institute for Infection and Immunity, Melbourne, VIC, Australia; i The National Centre for Infections in Cancer, Peter MacCallum Cancer Centre, Parkville, VIC, Australia

**Keywords:** Drug allergy, Low-risk, Risk assessment, Clinical decision rule, Antibiotics, Iodinated contrast media, Chemotherapy, Nonsteroidal anti-inflammatory drugs

## Abstract

Risk stratification in drug allergy implies that specific risk categories (eg, low, moderate, and high) classify historical drug hypersensitivity reactions. These risk categories can be based on reaction phenotypic characteristics, the timing of the reaction and evaluation, the required reaction management, and individual characteristics. Although a multitude of frameworks have been described in the literature, particularly for penicillin allergy labels, there has yet to be a global consensus, and approaches continue to vary between allergy centers. Immune-mediated drug allergies can sometimes be confirmed using skin testing, but a negative drug challenge is required to demonstrate tolerance and remove the allergy from the electronic health record (“delabel” the allergy). Even for quintessential IgE-mediated drug allergy, penicillin allergy, recent data reveal that a direct oral challenge, without prior skin testing, is an appropriate diagnostic strategy in those who are considered low-risk. Drug allergy pathogenesis and clinical manifestations may vary depending on the culprit drug, and as such, the optimal approach should be based on risk stratification that considers individual patient and reaction characteristics, the likely hypersensitivity reaction phenotype, the drug class, and the patient’s clinical needs. This article will describe low-risk drug allergy labels, focusing on β-lactam and sulfonamide antibiotics, nonsteroidal anti-inflammatory drugs, iodinated contrast media, and common chemotherapeutics. This review will also address practical management approaches using currently available risk stratification and clinical decision tools.

Drug allergies documented in electronic health records (EHRs)—drug allergy labels intended to document hypersensitivities, side effects, and intolerance—are mostly patient-reported reactions uncommonly verified by clinical evaluation or diagnostic testing.^1^ Reactions can be predictable non—immune-mediated (pharmacological) adverse drug reactions (eg, gastrointestinal symptoms and tachycardia) or unpredictable immune-mediated reactions (eg, anaphylaxis).^2^ Irrespective of whether these drug allergy labels are applied correctly or incorrectly, an “allergy” label can persist in the medical record over time and is rarely questioned.^3^ Drug allergy labels impede optimal clinical care across disciplines.^4–8^ β-Lactam antibiotic allergy labels carry substantial clinical, public health, and health-economic impacts.^9^

Immune-mediated drug allergies are investigated using skin prick and intradermal tests, followed by a drug challenge in those with prior skin test negativity. However, for some drug allergies, particularly penicillin allergy, the declining prevalence of confirmed immune-mediated drug allergies^10^ and the suboptimal diagnostic test performance^11^ have led to recent studies that reveal the safety of direct drug challenges (ie, provocation) in patients with a low-risk phenotype.^12^

This article will provide an overview of low-risk drug allergy labels in adults, focusing on key antibiotics, nonsteroidal anti-inflammatory drugs (NSAIDs), iodinated contrast media (ICM), and common chemotherapeutics that cause hypersensitivity. Further, we examine practical management approaches for patients with low-risk drug allergy labels using current risk stratification and clinical decision tools.

## LOW-RISK DRUG ALLERGY LABELS

Immune-mediated reactions to drugs most commonly manifest with cutaneous symptoms, although any organ can be affected.^13^ These skin manifestations are frequently benign and self-resolving generalized maculopapular exanthems or morbilliform drug eruptions.^14^ For delayed onset drug hypersensitivity reactions, early studies suggested a prevalence of 2% for cutaneous drug eruptions,^15^ with up to 90% of these being mild. Drug-induced urticaria, angioedema, and anaphylaxis have been described as immediate drug hypersensitivity mediated by IgE or non-IgE mechanisms. However, even confirmed true IgE-mediated reactions could be transient and lost over time, as described for penicillin and cephalosporin allergies.^16,17^ In the pediatric population, there is an increased prevalence of viral-induced reactions compared with true drug hypersensitivity. In this context, when children develop a morbilliform drug eruption or serum sickness—like reaction, this is uncommonly positive on rechallenge and assumed to have occurred as part of a viral infection or viral drug interaction.^18,19^ When the drug allergy is not verified in childhood, this label can be carried to the adult age.

The drug allergy history is a key aspect of a drug allergy evaluation.^20,21^ This allows the clinician to differentiate between a side effect and an immune-mediated reaction. Patient-reported allergy history, in conjunction with health record review, has facilitated clinical risk stratification and guided allergy diagnostic procedures and recommendations across clinical care settings for decades.^22–26^ More recently, the drug allergy history has been used to distinguish which patients may not need comprehensive allergy diagnostic testing. For example, in the context of penicillin allergy, clinical interviews, in the absence of skin testing, can assist in identifying very low-risk allergy phenotypes.^27,28^ Skin testing includes skin prick and intradermal testing (IDT), usually performed by allergists/immunologists or those with specialized training. Like any test with a low pretest probability, skin testing can lead to false positive results, which should be avoided in a low-risk population.^21^ A drug ingestion challenge remains the gold standard test to ensure that a patient can be successfully “delabeled.” Various health care professionals can safely perform low-risk drug challenges with consensus-driven or empirically derived low-risk definitions and adequate training and resources.^20,21^ When an allergy entry is removed, deleted, or inactivated from the EHR, it is termed delabeling.^29^

## PENICILLINS

Patient-reported penicillin reactions lead to increased medication errors, poor health delivery, and medical costs.^2^ The prevalence of penicillin allergy labels varies from less than 1% to more than 20%, depending on the population and geographical region.^30–32^

Among patients presenting with a suspected penicillin allergy, the most common symptoms are urticaria and maculopapular rashes, followed by angioedema, respiratory, and gastrointestinal symptoms (vomiting and diarrhea).^25,33^ However, benign, nonimmediate reactions (occurring >1 hour after exposure) to β-lactams are more commonly described than immediate reactions.^33–35^

### Risk stratification approach

**FIGURE 1.**
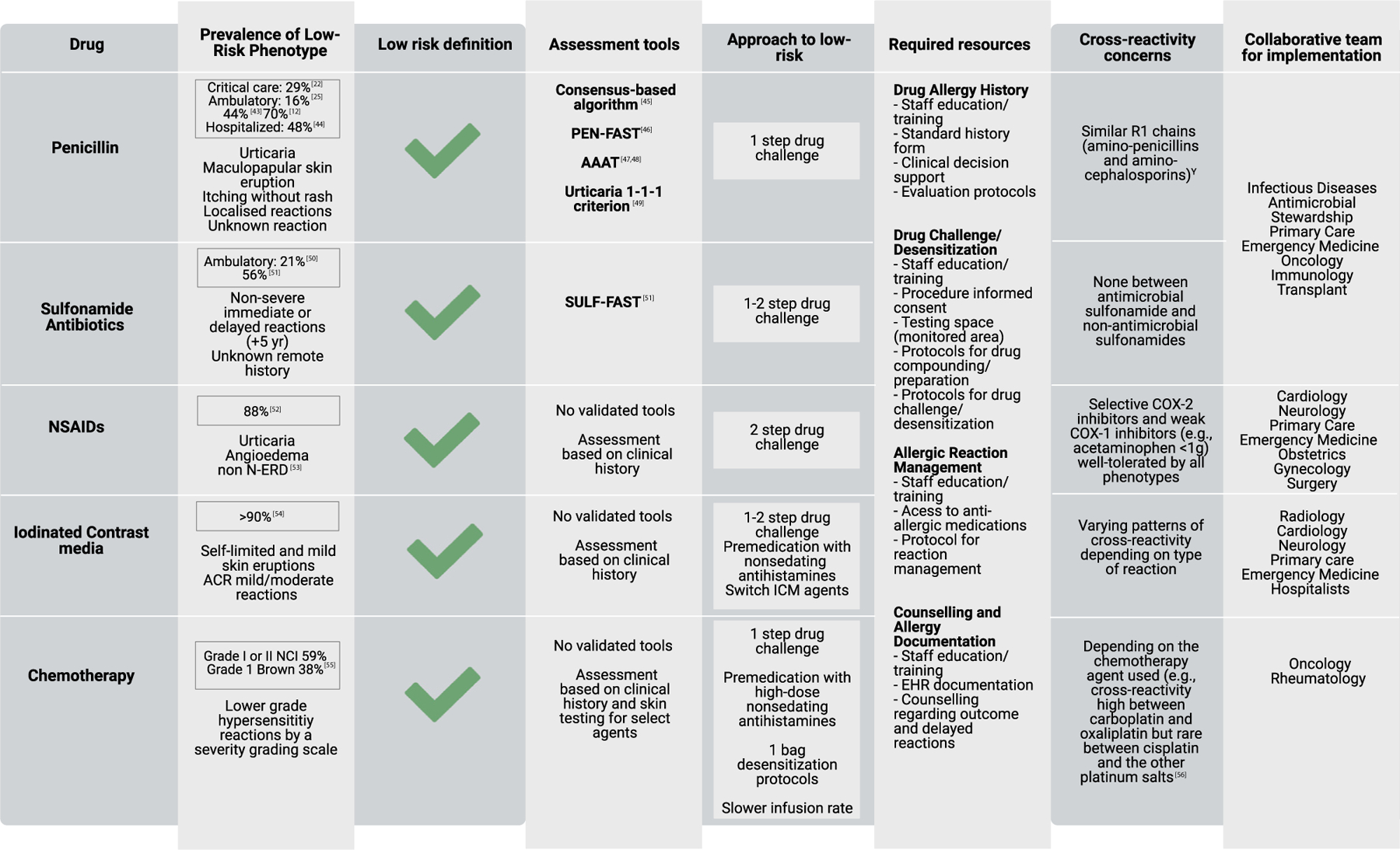
Low-risk drug allergy labels. ^Y^Aminopenicillins include amoxicillin, amoxicillin/clavulanate, ampicillin and aminocephalosporins include cephalexin, cefaclor, and cefadroxil. *AAAT*, Antibiotic Allergy Assessment Tool; *ACR*, American College of Radiology; *COX*, cyclooxygenase; *EHR*, electronic health record; *ICM*, iodinated contrast media; *NCI*, National Cancer Institute Common Terminology Criteria for Adverse Events scale; *N-ERD*, *NSAID*-exacerbated respiratory disease; *NSAID*, nonsteroidal anti-inflammatory drug.

Motivated by the declining rate of confirmed, immediate penicillin allergy in some centers^10^ (from >10% to <5%), the safety of drug challenges performed by allergists—severe reactions occurred in just 0.06% across 26,595 patients in 112 observational studies^36^—and the resource and logistical hurdles associated with performing skin tests,^26,37,38^ direct amoxicillin challenges were implemented initially by allergists in healthy individuals with low-risk histories.^34,39^ Today, direct drug challenges for low-risk β-lactam allergies, including penicillin, have been increasingly used in outpatient specialist settings,^25,40,41^ with 96% to 99% of low-risk penicillin allergies removed by an oral challenge.^22,23,32,42^ Depending on the settings and definitions, low-risk patients comprise 29% to 70% of patients with penicillin allergy labels that indicate a possible hypersensitivity (Figure 1).^12,22,25,43–56^

Statistical models have found that the absence of anaphylaxis, an unknown name of the index drug, a reaction occurring over a 1 year prior, and a reaction not being immediate (>1 hour from exposure) are associated with a lower risk.^41,46,57,58^ PEN-FAST was also derived using a regression model. In patients with a reported penicillin allergy (PEN), based on 4 allergy history criteria (time since reaction ≤5 years (F), anaphylaxis or angioedema (A), severe cutaneous adverse reaction (S), or whether pharmacologic treatment (T) was required for reaction), a PEN-FAST score of <3 is associated with 96.3% negative predictive value (NPV) (95% confidence interval [CI], 94.1%–97.8%).^46^

In a multicenter randomized controlled trial (RCT) across 6 outpatient specialized centers,^12^ 382 low-risk patients, defined by low PEN-FAST score,^46^ were assigned to either a direct oral challenge with penicillin (intervention arm) or penicillin skin testing followed by an oral challenge with penicillin for those who have negative skin tests (standard-of-care). The primary outcome of a physician-verified positive immune-mediated oral penicillin challenge was not different in the intervention group 1/187 (0.5%) compared with the control group 1/190 (0.5%).^12^ This trial further demonstrated that, in patients with a low-risk history, a direct oral penicillin challenge is a safe procedure to facilitate the removal of a penicillin allergy label.

Direct challenges have also been performed in the inpatient setting as part of delabeling initiatives or integrated β-lactam care pathways,^23,44,59,60^ and the effectiveness and safety of these procedures are comparable with those performed in the outpatient setting.^61,62^ Even in higher-risk individuals, in the critically ill intensive care unit setting, a prospective 2-year inpatient penicillin allergy delabeling study demonstrated that a direct amoxicillin challenge was tolerated in 99% of those considered with a low-risk penicillin allergy label.^23^ Only 2 cases (<1%) had a rash that led to the retention of the allergy label.^22^ In acutely infected individuals who require a β-lactam antibiotic other than amoxicillin, algorithm-driven direct challenges can also be performed with the indicated drug. Such algorithmic pathways have been implemented broadly in US hospitals across a variety of emergency and inpatient settings.^59,60,63–66^ Findings have included that this approach safely increased β-lactam antibiotic use and first-line therapy for specific infections,^26,67^ with no or minimal burden on consultants from allergy/immunology or infectious diseases.^64^ However, the approaches were most effective when combined with robust clinician education,^68,69^ and this approach is not a primary delabeling strategy. The American Academy of Allergy, Asthma & Immunology Drug Allergy Practice Parameter Update (2022) endorses consideration for a direct oral challenge to penicillin in adults with low-risk penicillin allergy histories.^29^ An interesting consideration is that most of the prospective validation studies that demonstrate the feasibility of direct oral challenges have been performed in countries with a similar penicillin allergy label burden, and there are few publications from other settings. For example, in a European setting, where the prevalence of penicillin allergy labels is lower,^30^ the reported percentage of delabeled individuals was also lower.^57,70^

### Drug allergy tools

Several penicillin allergy history-informed risk assessment algorithms have been suggested and are part of national and international guidelines.^29,71,72^ However, to our knowledge, algorithms were created largely through systematic literature review and expert consensus rather than through data derivation (Table I).

Shenoy et al^45^ led a consensus-based algorithm for risk-stratifying penicillin allergy that was reviewed and endorsed by 3 multidisciplinary US professional societies. It was intended for nonallergist use and has since been adapted and implemented in various settings, including routine hospital-based assessments and long-term care facility antibiotic stewardship toolkits.^81^ Non-allergists have also used the Antibiotic Allergy Assessment Tool,^47,48^ adapted for pediatric use^77^ and implemented in whole-of-hospital programs^75^ and rural antimicrobial stewardship programs.^82^

The PEN-FAST tool, externally validated in an RCT and international setting,^12^ includes items allergy specialists have always used for risk stratification in the allergy history but in a reduced and approachable format for nonallergists.^46,73^ Although the PEN-FAST score was not useful in children,^83^ it was useful in adults in the emergency department setting,^84^ pregnancy,^85^ and in a low- to middle-income country setting.^86^ Validations have yet to include diverse populations or geographic regions where the prevalence of reported penicillin allergy is lower^30^ or confirmed penicillin allergy is higher.^57,70^

An additional potential key predictor of a higher risk of confirmed penicillin allergy is immediate onset urticaria. Most predictive models derived to date have been underpowered to distinguish urticaria from other benign rash types.^87^ The urticaria 1-1-1 criterion, derived from 410 European patients, may be a helpful adjunctive tool for risk stratification to ensure that a patient with a cutaneous reaction history has a low-risk phenotype. The 1:1:1 criterion is urticaria within 1 hour after the first dose that had regressed within 1 day; its sensitivity and specificity are 85%, and negative and positive predictive values are 80% and 90%, respectively.^49^

## TRIMETHOPRIM-SULFAMETHOXAZOLE

Sulfonamide antibiotics are commonly prescribed for the immunocompromised population as prophylaxis and directed therapy for opportunistic pathogens such as *Pneumocystis jirovecii*.^88^ Sulfonamide antibiotics are the second most frequently reported antibiotic allergy,^89^ with 3% to 8% of patients reporting a sulfonamide allergy.^90^ Sulfonamide antibiotics can cause immediate or delayed hypersensitivity reactions with various reaction severities.^91^ Trimethoprim-sulfamethoxazole (TMP/SMX) is also one of the most prevalent causes of severe cutaneous adverse reactions, including Stevens-Johnson syndrome/toxic epidermal necrolysis and drug reaction eosinophilia and systemic syndrome.^92–94^

Although there is limited evidence on the cross-reactivity between sulfonamide antibiotics, it has been demonstrated that sulfonamide antimicrobials and sulfonamide nonantimicrobials are unlikely to cross-react due to differences in chemical structure,^51,95^ with the exception of sulfasalazine, a prodrug composed of 5-aminosalicylic acid linked to the sulfa antibiotic sulfapyridine.^96^ Sulfonamide antibiotic hypersensitivity reactions target N1 (heterocyclic ring) or N4 (an aromatic amine), absent in sulfonamide nonantibiotics.

### Risk stratification approach

Historically, patients with sulfonamide allergy histories consistent with a potentially immediate or benign delayed hypersensitivity were managed with desensitization or induction of tolerance procedures.^97^ Although skin testing with the nonirritant concentration was possible, it rarely yielded positive tests, and the test characteristics remain unknown.^95^ In patients with low-risk sulfonamide antibiotic allergies, a direct TMP/SMX challenge was tolerated in more than 94%.^50,98^ One- or 2-step TMP/SMX challenges have been described with up to 97% resulting in label removal in patients with low-risk allergy histories.^98,99^ However, it is unclear which patient populations may benefit from different allergy investigations or treatments. An analysis of older adults across 5 US sites recently found a lower 88% prevalence of sulfonamide antibiotic allergy delabeling after allergy testing.^100^ Interestingly, all 5 patients confirmed TMP/SMX allergic had immediate reactions observed in the office, with 1 individual receiving intramuscular epinephrine for progressive urticaria. Another consideration with sulfonamide antibiotic allergy delabeling is that most incident reactions are commonly delayed phenotypes. As such, drug challenge studies that have followed up delabeled patients with retreatment courses suggest that as many as 30% of patients may have delayed rashes with a full course.^98^ Therefore, all sulfonamide antibiotic allergy evaluations must include education and patient counseling regarding delayed reactions that can occur with the following treatment course.

### Drug allergy tools

In low-risk patients, a direct 1- or 2-step TMP/SMX oral challenge is well tolerated.^50,98^ SULF-FAST is a tool assessed using 2 international datasets^51^ from ambulatory specialist care.^46,50^ SULF-FAST could identify individuals at low risk for a true allergy who could proceed to an oral challenge as a delabeling strategy. A 1-step challenge was recommended for most patients with nonanaphylaxis and non-SCAR or a SULF-FAST score of less than 3. While specificity and NPV were high, sensitivity was lower, particularly in the US dataset (38.5%).^51^

## NONSTEROIDAL ANTI-INFLAMMATORY DRUGS

After antibiotics, NSAIDs are the second most common drug class causing hypersensitivity reactions, described in up to 3.5% of the population.^89^ NSAIDs may induce a variety of reactions, although most reactions occur within 6 hours and are cutaneous (eg, urticaria and angioedema). However, systemic reactions such as severe anaphylaxis^101^ and delayed skin reactions (eg, fixed drug eruptions or Stevens-Johnsons syndrome^102,103^) have also been reported from NSAIDs.

Classification of NSAID-induced reactions is based on clinical history (eg, reaction timing and characteristics), presence of comorbid conditions, and drug challenges. NSAID-induced urticaria/angioedema/anaphylaxis (NIUAA) is the most common phenotype, representing >40% of reported reactions in adults.^101^ Symptoms are typically elicited by potent cyclooxygenase-1 (COX-1) inhibitors, with isolated cutaneous symptoms reported most commonly; however, 9% to 28% of adults may report reactions consisting of blended features with symptoms or signs extending outside of the skin or anaphylaxis.^101,104^ Up to 30% of patients with chronic spontaneous urticaria (CSU) experience an acute exacerbation of their disease after NSAID exposure^53,105^ (NSAID-exacerbated cutaneous disease [NECD]). Notably, patients with NECD may tolerate NSAIDs during CSU remission or clinical control.^106^ Patients with underlying asthma, chronic rhinosinusitis, and nasal polyposis may react with acute respiratory reactions to COX-1 inhibitors in the context of aspirin- or NSAID-exacerbated respiratory disease (N-ERD). As with the other cross-reactive phenotypes, including NIUAA and NECD, patients with N-ERD can report reactions to chemically nonrelated NSAIDs.

Less commonly, some patients may develop immediate reactions (often within an hour) to a single NSAID^107,108^ or structurally related NSAIDs (single NSAID-induced urticaria/angioedema/anaphylaxis [SNIUAA]). These reactions are thought to be IgE-mediated, and no cross-reactivity with other NSAIDs is expected. Although 1 study did observe cross-reactivity in some patients with reactions induced by more than 1 drug within the class of propionic acid derivatives, it is also important to note that most patients with SNIUAA in this same study demonstrated tolerance of structurally related NSAIDs.^109^

Separately, NSAIDs can act as cofactors to aggravate hypersensitivity reactions to foods (NSAID-exacerbated or -induced food allergy), and patients may be misdiagnosed with NSAID hypersensitivity; for these patients, a food rather than drug allergy workup should take precedence.^110^

### Risk stratification approach

Most patients with NSAID allergy histories can tolerate NSAIDs. Several studies of patients with prior NSAID reactions have found that 79% to 86% of patients with an NSAID label and referred to a drug challenge by allergists tolerate NSAIDs. However, these patients had no history potentially consistent with N-ERD.^53,111^ On the contrary, drug challenges are positive in 86% to 100% of patients reporting prior NSAID reactions consistent with N-ERD,^112^ and as such, these procedures should only be performed when there is diagnostic uncertainty in clinics or hospitals experienced in managing potential reactions.^21^

The gold standard for diagnosing NSAID hypersensitivity is a drug challenge. Skin testing has no role; it is not standardized nor recommended.^21^ Given the variable phenotypes and their complexity, risk stratification approaches have been entirely within the allergy specialist domain. NSAID drug challenges are considered whenever possible for appropriate non-N-ERD phenotype candidates. Although protocols vary widely,^113^ a study of 249 individuals demonstrated the safety of a 2-step drug challenge for all non-N-ERD NSAID hypersensitivity.^53^ Although no global consensus^113–115^ currently exists, these data indicate that a 2-step direct oral challenge protocol can be feasibly and safely performed in the outpatient specialist setting to evaluate non-N-ERD phenotypes.

Selective COX-2 inhibitors (ie, celecoxib) and weak COX-1 inhibitors (ie, acetaminophen at <1 g/d)^116^ rarely provoke reactions in cross-hypersensitive patients and can typically be taken safely by patients with suspected or confirmed NSAID hypersensitivity^21,115,117^ of any phenotype. In some cases, for example, a severe prior NSAID-induced reaction, the first administration can be performed via a drug challenge under allergist supervision.

The recommendation in patients with NSAID-induced urticaria and angioedema is to proceed with a direct oral challenge with aspirin to evaluate for cross-reactivity to alternate NSAIDs. For patients with a history of non-N-ERD aspirin hypersensitivity and an acute need for aspirin for cardiovascular disease, a 2-step aspirin challenge is suggested.^21^

### Drug allergy tools

No clear risk stratification tools have been published for NSAID allergy. However, several groups identified individual risk factors associated with a positive NSAID challenge,^53,111^ such as younger age (<40 years), male gender, recent prior reaction, prior immediate reaction or cross-reactive NSAID hypersensitivity history, or comorbid chronic spontaneous urticaria. Although no consensus-based algorithm exists, when the allergy history is inconsistent with N-ERD, a drug challenge (ie, with a 2-step protocol) should be strongly considered.^21^ Non-N-ERD patients comprise most patients presenting for NSAID allergy (88%).^52^

## IODINATED CONTRAST MEDIA

ICM is associated with predictable nonimmune-mediated (pharmacological) reactions but also immediate (<1 hour of administration) and delayed hypersensitivity reactions.^118^ The incidence of reactions to ICM, including severe, life-threatening reactions, has decreased to 0.2% to 0.7% since the use of low-osmolarity contrast agents.^116,119–121^ Most of these reactions are self-limited and mild, such as localized skin eruptions, and do not require medical intervention or premedication protocols.

Immediate reactions to ICM have largely been considered noneIgE-mediated and therefore have classically been managed with pretreatment regimens that include oral corticosteroids and H1-antihistamines.^122^ However, the 2020 Anaphylaxis Practice Parameter recommends against routine corticosteroid or antihistamine administration to prevent recurrent immediate reactions in patients with a history of ICM hypersensitivity.^123^ In addition, the routine use of corticosteroids has negative implications for patients, particularly those with active infections or diabetes. Some studies have reported a significant impact of antihistamine premedication alone (ie, without corticosteroids) for reducing immediate reactions in those with a prior history of ICM reaction. In one study of 196,081 patients, including 570 who had prior reactions to ICM, premedication with antihistamine (odds ratio [OR]: 0.53 [95% CI: 0.33, 0.86]) and switching contrast agents (OR: 0.51 [95% CI: 0.36, 0.73]) were associated with reduced odds of recurrent immediate reactions.^124^ In cases where the pretest probability is low, switching agents increased the likelihood of receiving a nonecross-reactive ICM, and previous reports have shown a benefit for this pragmatic strategy. Allergy consultation may be advisable in cases where the pretest probability is high, given that prior studies demonstrated that premedication protocols do not prevent all severe recurrent reactions or cases of anaphylactic shock.^125^

For delayed reactions occurring from day 1 through 7, antihistamine premedication but not changing the agent was associated with a reduced risk of recurrence.^54^ As such, optimal management might include switching contrasts and offering nonsedating antihistamine premedication for individuals who present with low-risk ICM reactions. Still, it is common radiology practice for patients with contrast reactions to receive antihistamine and corticosteroid premedication,^126^ typically the Greenberger protocol.^122^

Skin testing has possible benefits for a subset of higher-risk, well-phenotyped immediate and delayed reactions.^125,127–129^ Indeed, skin testing can help confirm a specific hypersensitivity and guide safe ICM rechallenge with a non—cross-reactive agent.^118,125,130,131^ However, IDT does not predict future reactions,^132^ and sensitivity and specificity are suboptimal. The main benefit of skin testing is likely for patients who present with higher-risk or recurrent ICM reactions, as this specialized testing could support which, if any, alternative ICM can be safely used.^118,130^

### Risk stratification approach

The risk stratification for ICM closely relies on the initial reported reaction to the product. Based on this, reactions can be classified as mild, moderate, or severe, as described by the American College of Radiology.^126^ Patients with mild and moderate reactions should be readministered the culprit or alternative ICM after a risk-benefit discussion. To reduce the risk of recurrent reactions, interventions such as switching contrast agents or premedicating with high-dose nonsedating antihistamines can be considered. Skin tests have a role for the highest-risk patients and those with breakthrough reactions.^130^ A monitored ICM challenge has been performed in various settings to confirm tolerance.^118,133^ Fortunately, the vast majority of reactions presenting similarly to hypersensitivity reactions are low-risk non—IgE-mediated phenotypes.^54^

## CHEMOTHERAPY

Hypersensitivity reactions to chemotherapy are often not explained by the pharmacologic mechanism of action of the medication.^134^ Although immediate and delayed reactions are reported to chemotherapeutic agents, largely platins and taxanes,^135^ the most common reactions are probably infusion related, presenting with flushing, pruritus, hemodynamic changes, shortness of breath, throat or chest tightness, gastrointestinal symptoms, chest, back, or abdominal pain, fever, chills, seizures, dizziness, and syncope.^136,137^ Phenotypes of platin hypersensitivity reactions include IgE-mediated reactions, cytokine release reactions, and mixed reactions.^89^ Taxanes may cause mast cell and/or basophil activation through IgE-mediated mechanisms, direct action on basophils, or IgG-mediated mechanisms that cause complement activation and release of anaphylatoxins.^138,139^

The repetitive administration of platinum-based chemotherapy agents, particularly after a break in treatment, can lead to hypersensitivity reactions (up to 27% after 7 doses).^140^ Allergy to taxanes, however, typically occurs during the first dose.^141^ Hypersensitivity reactions to chemotherapeutics prevent patients from receiving the most effective treatments, particularly in sites without allergist access or desensitization protocols.^8^

### Risk stratification approach

Risk has typically been defined not by patient-reported historical symptoms but by reaction severity, as the reaction is typically observed by a health care professional permitting severity grading. Low risk includes the lower grades from Ring and Messmer,^142^ Brown,^143^ or National Cancer Institute Common Terminology Criteria for Adverse Events^144^ scales (approximately one-third to one-half of reactions are lower grades^55^). Skin test results, particularly for the platinum agents and serum tryptase assessments, have also informed risk stratification. As for other suspected drug anaphylaxis-type reactions, serum tryptase is performed 30 minutes to 1.5 hours after the acute reaction. A baseline tryptase is also performed at least 24 hours after the acute reaction. The platinum-based skin testing^145,146^ may guide whether a desensitization protocol is necessary.^147,148^ The most common desensitization protocols are 3-bag, serial 10-fold dilutions that result in a stepwise increase to promote tolerance. Recently, shorter desensitization protocols have emerged, particularly for those considered low-risk, with 1 bag protocols from as few as 4 to as many as 17 steps.^149^ Studies have demonstrated comparable safety of 1 bag protocols with reduced patient time and expended resources.^55,149^

If the reaction is limited to mild or moderate symptoms, direct rechallenge may also be possible, restarting with a slower infusion rate of the drug that may allow treatment continuation.^150^ Drug challenges may also reduce the number of desensitizations performed. In one large study, the use of a drug challenge for all patients with reactions to platins, taxanes, and biologics permitted delabeling, or excluding allergy, in 44% (229 of 515).^151^ Subsequently, only those with a confirmed allergy who reacted to the drug challenge required desensitization. However, there is currently no agreed-upon definition for what defines a low-risk allergy label for chemotherapeutics, and there is a wide variation with respect to the availability to perform chemotherapeutic skin testing, desensitization, and drug challenge.^152^

Depending on the severity grade of the reaction and the culprit chemotherapy agent, several risk stratification approaches have been suggested to determine if a challenge or a desensitization protocol is required. For example, using the Brown classification for anaphylaxis,^143^ one can determine the management approach for taxane reintroduction.^153^ The role of skin testing in this context is unknown. However, fewer skin tests and even omitting skin test steps have been tried to reduce the burden of testing and required visits in this cancer population. The only recommended options for nonimmediate reactions without a convincing history are a slower infusion rate or increasing antihistamine or corticosteroid premedication but not desensitization.^137,154^

## CONCLUSION AND FUTURE DIRECTIONS

Allergy history-based evaluation and risk stratification tools can assess patients across all common drug allergy classes. Given the high prevalence of drug allergy labels and low rate of drug hypersensitivity confirmation, it is essential to identify those who are low-risk, as these individuals may be able to continue their care without an allergy specialist evaluation with the use of standardized collaborative protocols for rechallenge or desensitization. We recommend that all hospitals and ambulatory centers work with allergy specialists to consider low-risk approaches for each drug category to optimize the quality and safety of care (Figure 1). Low-risk patients and patients with non—immune-mediated reactions could be evaluated outside the allergy setting or with telehealth (e-consults) guiding care. Then, allergists could focus on the patients reporting moderate/high-risk reactions (eg, PEN-FAST more than 3) or higher-risk hosts (eg, patients with cystic fibrosis on home oxygen, pregnancy, pending bypass surgery, and hemodynamically unstable).

Any collaborative approach that includes nonallergists should include clear protocols, informed consent training, allergy record documentation training,^155^ and anaphylaxis training.^156,157^ All clinical sites where drug challenges or desensitizations are supervised should be equipped to diagnose and treat anaphylaxis. Finally, standardized recommendations for patients should be provided regarding the risk of reactions in the future. Patients with a reaction after delabeling should be instructed to document their reaction and seek evaluation by allergy/immunology.

There has been a recent paradigm shift in drug allergy, whereby allergy specialist assessment may not be necessary for all low-risk drug allergy patients, particularly for the low-risk penicillin allergyelabeled patient. Additional work is required to identify the best approach for non—β-lactam antibiotics, NSAIDs, contrast agents, and chemotherapeutics.

## Figures and Tables

**TABLE I.** Penicillin allergy risk assessment tools

Author/country	Primary derivation	User group	Population	Assessment outcomes	Sensitivity (%)	Specificity (%)	NPV (%)	PPV (%)	External validation or usage	Special populations
Sabato et al 2021 (Europe)^49^	Retrospective-multivariable logistic regression	Allergists	N = 410	1-1-1 criterion (1 urticarial eruption within the first hour and after the first dose)	85	85	80	90	n/a	n/a
Stevenson et al 2020 (AUS)^41^	Retrospective-multivariable logistic regression	Allergists	N = 447	Low-risk criteria: non-SCAR rash or rash without angioedema and >1 year previously	80	61	97	n/a	n/a	n/a
Trubiano et al 2020 (AUS/US)^46^	Prospective data-multivariable logistic regression	Clinicians	N = 622 (primary validation) N = 945 external validation)	3-point clinical criteria (maximum score 5) Low/moderate/high Low-risk criteria: PEN-FAST score <3	71	79	96	25	USA/Canada/Australia^12^ France^73^ (retrospective)	n/a
Moreno et al 2020 (Spain)^74^	Retrospective (R) logistic regression prospective (P) data	Clinicians	N = 656 (R) N = 615 (P)	Artificial neural network (ANN)*	90 (R) 81 (P)	86 (R) 98 (P)	92 (R) 95 (P)	82 (R) 91 (P)	n/a	n/a
Siew et al 2019 (UK)^58^	Retrospective-multivariable logistic regression	Allergists	N = 1092	Low-risk criteria: no anaphylaxis, reaction >1 year, unknown index drug	n/a	n/a	98	n/a	n/a	n/a
Devchand et al 2019 (AUS)^47^	Expert opinion	Pharmacists Doctors Nurses	Adult inpatients/outpatients	Low-risk criteria: childhood rash, MPE >10 years, unknown >10 years	92	94	n/a	n/a	AUS^32,48,75,76^	Pediatric^77^
Shenoy et al 2019 (US)^45^	Expert opinion^†^	Clinicians	Not specified	Low-risk criteria: type A, pruritus without rash, unknown reactions >10 years without IgE features, family history	n/a	n/a	n/a	n/a	USA^78,79^ Canada^79^	General practice^80^ Long-term care facilities^81^
Chiriac et al 2018 (France)^57^	Retrospective-multivariable logistic regression	Allergists	N = 1991 (retrospective) N = 200 (prospective)	5-point criteria No validated low risk	51	75	83	40	n/a	n/a

Low risk was defined as an assessment outcome that led to or recommended a direct oral penicillin challenge.

*MPE*, maculopapular exanthem; *n/a*, nonapplicable; *NPV*, negative predictive value; *PPV*, positive predictive value; *SCAR*, Severe Cutaneous Adverse Reaction.

* Factors considered were age, time since reaction, culprit antibiotic, route administration, treatment day, latency period, type of reaction, episode duration, and total IgE.

† Approved and endorsed by the American Academy of Allergy, Asthma & Immunology, Infectious Diseases Society of America, and Society for Healthcare Epidemiology of America.

## INFORMATION FOR CATEGORY 1 CME CREDIT

Credit can now be obtained, free for a limited time, by *reading* the review articles in this issue. Please note the following instructions.

### Method of Physician Participation in Learning Process:

The core material for these activities can be read in this issue of the Journal or online at the *JACI: In Practice* Web site: www.jaci-inpractice.org/. The accompanying tests may only be submitted online at www.jaci-inpractice.org/. Fax or other copies will not be accepted.

### Date of Original Release:

May 1, 2024. Credit may be obtained for these courses until April 30, 2025.

### Copyright Statement:

Copyright © 2024–2026. All rights are reserved, including those for text and data mining, AI training, and similar technologies.

### Overall Purpose/Goal:

To provide excellent reviews on key aspects of allergic disease to those who research, treat, or manage allergic disease.

### Target Audience:

Physicians and researchers within the field of allergic disease.

### Accreditation/Provider Statements and Credit Designation:

The American Academy of Allergy, Asthma & Immunology (AAAAI) is accredited by the Accreditation Council for Continuing Medical Education (ACCME) to provide continuing medical education for physicians. The AAAAI designates this journal-based CME activity for 1.00 *AMA PRA Category 1 Credit*^™^. Physicians should claim only the credit commensurate with the extent of their participation in the activity.

### List of Design Committee Members:

Ana Maria Copaescu, MD, FRCPC, Lily Li, MD, Kimberly G. Blumenthal, MD, MSc, Jason A. Trubiano, MBBS, BBiomedSci, PhD, FRACP (authors); David A. Khan, MD (editor)

### Learning objectives:

1. To understand the clinical impact of low-risk drug allergy labels on patient care.
2. To identify risk stratification approaches and diagnostic tools for low-risk drug allergy.
3. To understand the limits of currently available diagnostic tools in managing low-risk drug allergies.

### Recognition of Commercial Support:

This CME has not received external commercial support.

## Abbreviations used

CI: Confidence interval
COX-1/2: Cyclooxygenase-1/2
CSU: Chronic spontaneous urticaria
EHR: Electronic health record
ICM: Iodinated contrast media
IDT: Intradermal testing
NECD: Nonsteroidal anti-inflammatory drug—exacerbated cutaneous disease
NIUA: Nonsteroidal anti-inflammatory drug—induced urticaria/angioedema
NPV: Negative predictive value
NSAID: Nonsteroidal anti-inflammatory drug
OR: Odds ratio
RCT: Randomized controlled trial
SNIUAA: Single nonsteroidal anti-inflammatory drug—induced urticaria/angioedema, anaphylaxis, or both
TMP/SMX: Trimethoprim-sulfamethoxazole
